# Prevalence and risk factors of vitamin D deficiency among patients with chronic myofascial pain syndrome: a cross-sectional study

**DOI:** 10.1186/s40795-023-00792-z

**Published:** 2023-11-14

**Authors:** Pimpitcha Channarong, Chanwit Phongamwong

**Affiliations:** Department of Rehabilitation Medicine, Phramongkutklao Hospital, Phramongkutklao College of Medicine, Bangkok, Thailand

**Keywords:** Prevalence, Risk factors, Vitamin D deficiency, Myofascial pain syndrome, Cross-sectional studies

## Abstract

**Background:**

Myofascial pain syndrome (MPS) is a common muscle condition characterized by painful trigger points. Vitamin D deficiency has been recognized as a precipitating factor of MPS. The present study aimed to determine the prevalence and risk factors of vitamin D deficiency in patients with chronic MPS.

**Methods:**

A cross-sectional study was conducted, using a structured face-to-face interview to collect demographic information, clinical characteristics, pain duration and location, as well as the bodily pain subscale of SF36 and EQ-5D-5 L. The Elecsys vitamin D total II assay was used to measure serum total 25-hydroxyvitamin D level.

**Results:**

Of 120 participants, vitamin D insufficiency (20 to 29.9 ng/ml) and deficiency (< 20 ng/ml) were 47.5% (95% CI: 38.3–56.8%) and 34.2% (95% CI: 25.8–43.4%), respectively. The adjusted odds ratios for vitamin D deficiency of participants aged < 45 years and who reported having ≤ 15 min sunlight exposure per day were 3.5 (95% CI: 1.54 to 7.98) and 2.38 (95% CI: 1.05 to 5.26), respectively. The bodily pain score (r = − 0.02, P = 0.86) and EQ-5D-5 L utility (r = 0.04, *P* = 0.66) did not significantly correlate with vitamin D levels.

**Conclusion:**

Approximately one third of patients with chronic MPS had vitamin D deficiency. Age < 45 years and sunlight exposure ≤ 15 min/day were identified as potential risk factors for vitamin D deficiency in MPS patients.

## Background

Vitamin D is an essential nutrient mainly synthesized endogenously with sunlight exposure [1]. It has a significant role in the metabolism of bone and skeletal muscle. The active form of vitamin D (1,25-dihydroxyvitamin D, Calcitriol) activates vitamin D receptors (VDR) in various tissues, including the musculoskeletal system, which are primarily responsible for regulating cell growth and cell differentiation [2, 3]. There is biological evidence that vitamin D deficiency negatively affects skeletal muscle function through oxidative stress and mitochondrial dysfunction [4]. Vitamin D also acts as a neuroactive steroid that modulates neuronal excitability in the pain pathway [1]. Hence, vitamin D deficiency may result in a central neuronal hypersensitivity state associated with chronic pain.

Vitamin D deficiency, determined as a serum 25-hydroxyvitamin D level < 20 ng/mL (50 nmol/L) [5], is a common public health concern in many countries, including those with relatively high natural sun exposure [6]. In temperate zones such as the US, Canada, and Europe, the estimated overall prevalence rate of vitamin D deficiency has been reported as 37–42% [7–9], while in tropical zones such as South East Asian countries, the prevalence among workers in Thailand and Singapore have been reported as 22% and 33%, respectively [10, 11]. The main risk factors linked to low serum vitamin D levels were inadequate exposure to sunlight and intake of vitamin D-rich foods, as well as obesity, dark skin tone, and diabetes mellitus [12].

Vitamin D deficiency appears to be more prevalent among patients with chronic musculoskeletal pain. For instance, a cross-sectional study in the US reported that 93% of 150 patients with persistent musculoskeletal pain had vitamin D deficiency [12]. A large cross-sectional study in Turkey found that 74% of 14,925 patients with widespread musculoskeletal pain had vitamin D deficiency [13]. Furthermore, 83% among 299 patients with chronic low back pain in Saudi Arabia had vitamin D deficiency [14].

Myofascial pain syndrome (MPS) is a common pain disorder with myofascial trigger points. It has a prevalence rate of up to 93% among patients with nonspecific musculoskeletal pain [15]. Vitamin D deficiency has been clinically recognized as a precipitating factor indicative of MPS [16, 17]. Moreover, an observational study in Turkey reported that the prevalence of vitamin D deficiency among 120 patients with MPS was 82% [17]. However, in Thailand, no epidemiologic studies have been conducted on this issue among patients with MPS. Therefore, the present study primarily aimed to determine the prevalence and associated risk factors of vitamin D deficiency in Thai adults with chronic MPS. Furthermore, the relationship between vitamin D level and quality of life was evaluated.

## Methods

### Study design and subjects

This study had a cross-sectional design and was conducted at the Department of Rehabilitation Medicine, Phramongkutklao Hospital, Thailand, between July 2020 and November 2022. Inclusion criteria included Thai adults (18 to 70 years) who were diagnosed with chronic MPS (> 3 months). Myofascial trigger points were defined by at least two of the following criteria: a taut band, a hypersensitive spot, and referred pain [18]. The current study excluded patients who (1) received vitamin D supplements within 3 months before enrolment, (2) had a history of long-term opioid use, (3) had other neurological diseases such as stroke, myelopathy, and radiculopathy, (4) had structural deformities such as scoliosis or discrepancy in limb length (LLD), (6) had comorbidities that interfered with vitamin D absorption and metabolism such as osteoporosis, chronic kidney or liver diseases, and use of anticonvulsants and glucocorticoids. After explanations and discussions of the study protocol, potential subjects provided written informed consent for study participation. The Institutional Review Board of the Royal Thai Army Medical Department approved this study protocol (document identifier: R071q/64_Exp).

### Data collection

Demographic and clinical characteristics of the subjects were collected using a structured face-to-face interview. Sociodemographic characteristics included age, occupation, sun exposure time, smoking status, physical exercise regularity, and sunscreen use. Previous treatments including dry needling, physical therapies, and medications were recorded. The duration and location (axial and non-axial) of pain were ascertained. The bodily pain subscale Short Form 36 (SF-36), scored from 0 (worst) to 100 (best), was also assessed [19]. Furthermore, the Thai version of EuroQol 5-Dimension, 5-Level (EQ-5D-5 L) was evaluated, which is a practical, reliable, valid, and responsive questionnaire to assess the quality of life in Thai patients with chronic diseases [20]. The answers given to EQ-5D-5 L were converted into health utility scores ranging from 0 (death) to 1 (perfect health).

### Measurement of vitamin D level

Blood samples were taken from each participant after their interview at the Phramongkutklao Hospital clinical laboratory. Serum 25-hydroxyvitamin D2 and D3 concentration was analyzed using an Elecsys vitamin D total II assay (Roche Diagnostics) that agrees excellently with the reference measurement method (liquid chromatography tandem mass spectrometry) [21]. Vitamin D status was classified as deficiency (< 20 ng/ml), insufficiency (20 to 29.9 ng/ml), and sufficiency (≥ 30 ng/ml) [22].

### Statistical analysis

An estimated sample size of 101 participants was required based on the formula *n = z*^*2*^_*α/2*_*p(1-p)/d*^*2*^. An alpha level of 0.05 and an estimated precision (d) of 0.05 were selected. The estimated *p* was 0.93 based on the study of Plotnikoff and Quigley (2003) [12].

To identify risk factors associated with vitamin D deficiency (0 = at least 20 ng/mL, 1 = less than 20 ng/mL), Chi-square tests were used for a univariable analysis. Then, all variables was analyzed using binary logistic regression with backward stepwise (pr = 0.25 and pe = 0.2). Pearson correlation coefficient (r) was used to determine relationship between vitamin D levels and SF-36 bodily pain score or EQ-5D-5 L health utility score. A *P*-value < 0.05 necessitated rejecting the null hypothesis, which is interpreted as statistically significant.

## Results

A total of 120 subjects (67 female, 53 male) participated in this study. Before their involvement in the study, 107 subjects (89.2%) had undergone various treatments. Among the treatments received, dry needling to release myofascial trigger points was the most commonly reported (99 subjects, 82.5%). The prevalence of vitamin D insufficiency and deficiency were 47.5% (95% CI: 38.3–56.8%) and 34.2% (95%CI: 25.8–43.4%), respectively (Table 1). The average values (standard deviation) of the vitamin D level among participants with sufficient, insufficient, and deficient status were 35.1 (4.5), 24.1 (2.7), and 16 (2.8) ng/ml, respectively.

**Table 1:** Prevalence of vitamin D insufficiency and deficiency

Vitamin D status	Frequency	Percentage	95% Confidence interval
Sufficiency	22	18.3%	11.9–26.4%
Insufficiency	57	47.5%	38.3–56.8%
Deficiency	41	34.2%	25.8–43.4%

**Fig. 1:**
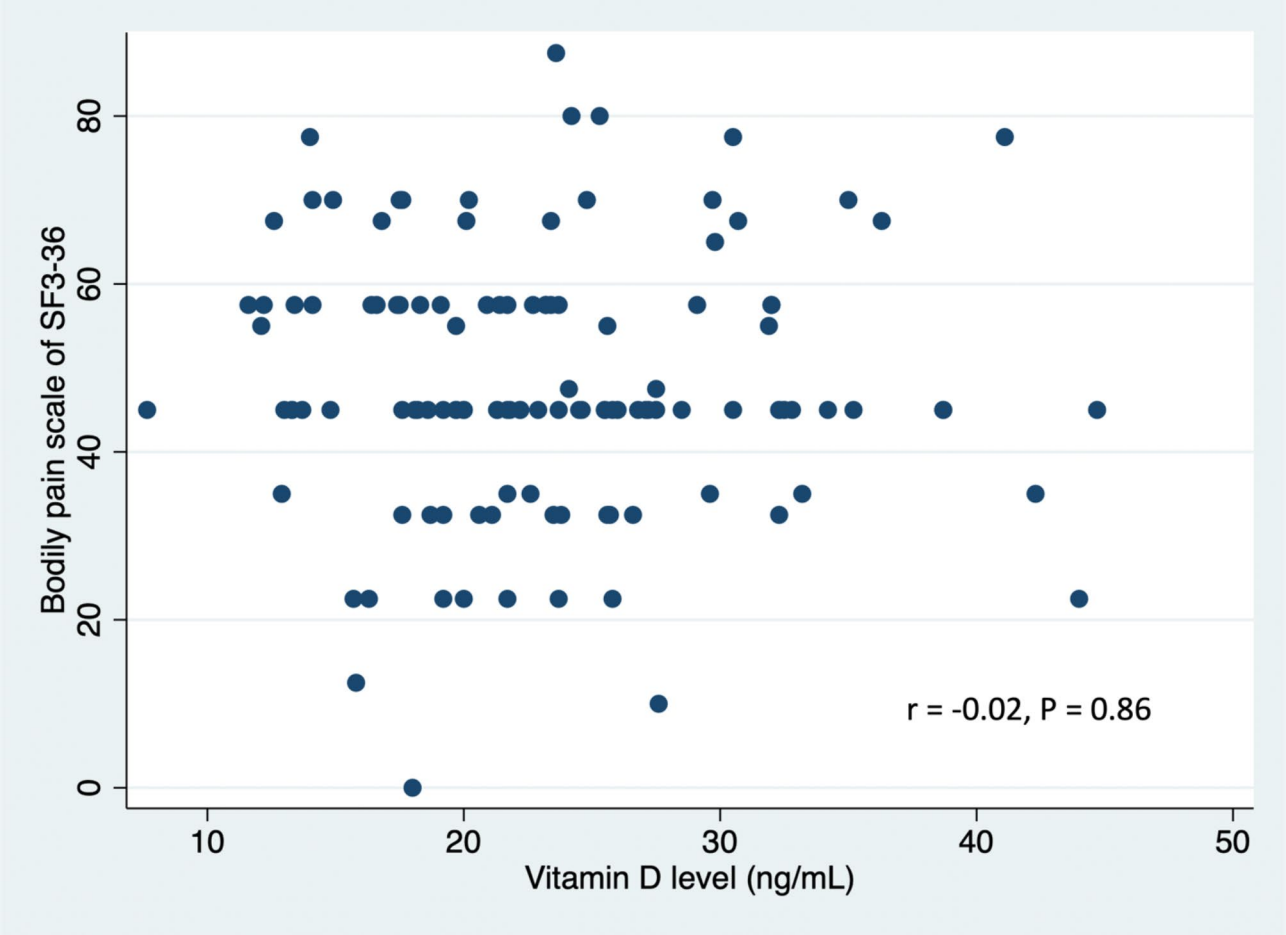
Scatter plot showing correlation between bodily pain scale (SF3-36) and vitamin D levels

**Fig. 2:**
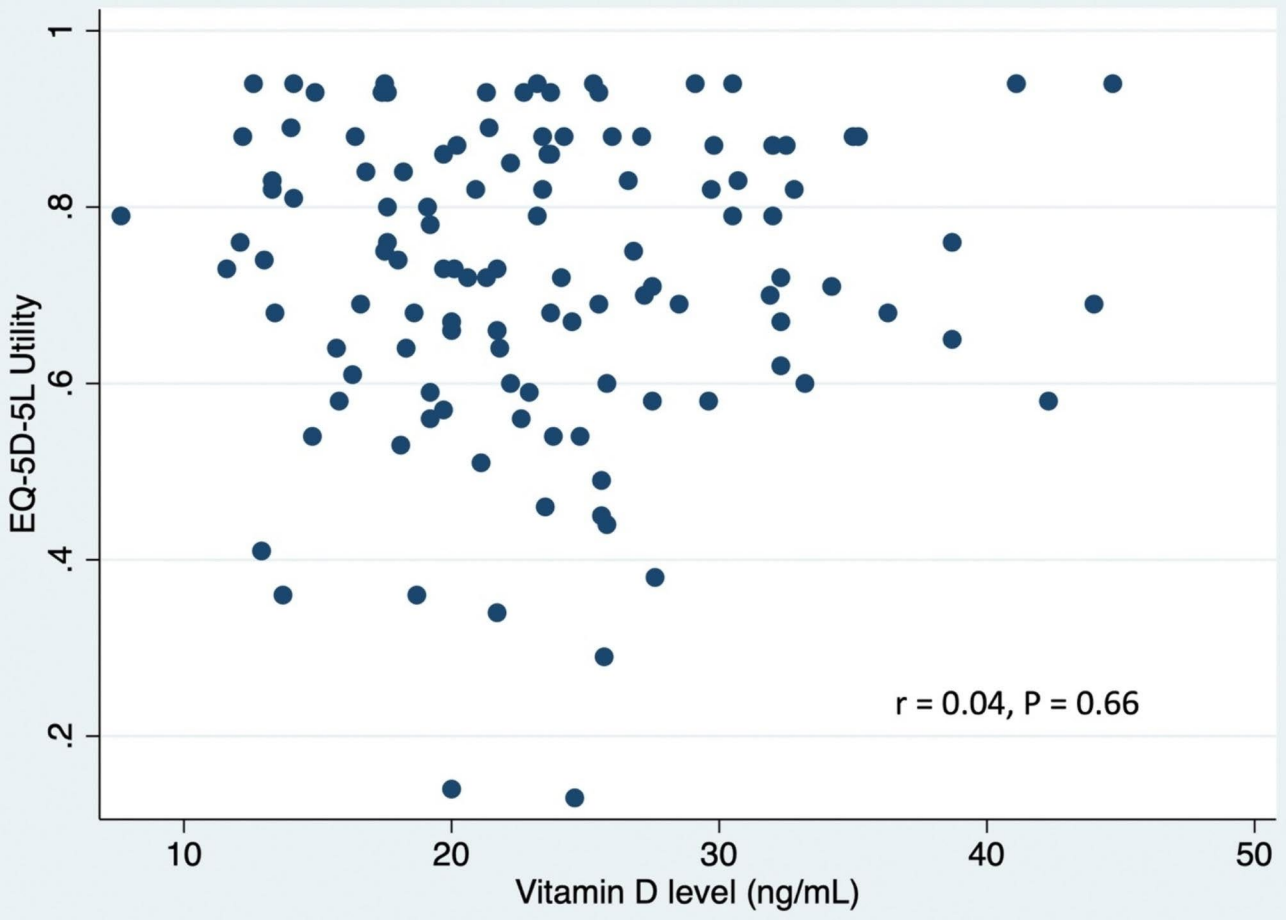
Scatter plot showing correlation between health utility (EQ-5D-5 L) and vitamin D levels

Only the age group (P = 0.005) and sunlight exposure (P = 0.04) showed a statistically significant association with vitamin D deficiency in the crude analysis (Table 2). From the logistic regression model, the adjusted odds ratio of age < 45 years and sunlight exposure ≤ 15 min/day were 3.5 (95% CI: 1.54 to 7.98) and 2.38 (95% CI: 1.05 to 5.26) as shown in Table 3. Additionally, there was no correlation between vitamin D level and SF-36 bodily pain score (r = − 0.02, P = 0.86) or EQ-5D-5 L health utility score (r = 0.04, P = 0.66), as depicted in Figs. 1 and 2.

**Table 2:** Association between demographic data and vitamin D deficiency

Variable	Overall	Vitamin D deficiency		*P*-value
		Yes	No	
Age				
< 45 years	52 (43.3)	25 (48.1)	27 (51.9)	0.005**
≥ 45 years	68 (56.7)	16 (23.5)	52 (76.5)	
Sex				
Male	53 (44.2)	16 (30.2)	37 (69.8)	0.41
Female	67 (55.8)	25 (37.3)	42 (62.7)	
BMI				
< 25 kg/m^2^	81 (67.5)	28 (34.6)	53 (65.4)	0.89
≥ 25 kg/m^2^	39 (32.5)	13 (33.3)	26 (66.7)	
Hypertension				
Yes	35 (29.2)	13 (37.1)	22 (62.9)	0.66
No	85 (70.8)	28 (32.9)	57 (67.1)	
Diabetes mellitus				
Yes	18 (15)	5 (27.8)	13 (72.2)	0.54
No	102 (85)	36 (35.3)	66 (64.7)	
Workplace				
Indoor	110 (91.7)	39 (35.5)	71 (64.5)	0.49
Outdoor	10 (8.3)	2 (20)	8 (80)	
Location of pain				
Axial	94 (78.3)	34 (36.2)	60 (63.8)	0.62
Non-axial	10 (8.3)	2 (20)	8 (80)	
Both	16 (13.4)	5 (31.3)	11 (68.8)	
Duration of pain				
< 12 months	39 (32.5)	14 (35.9)	25 (64.1)	0.78
≥ 12 months	81 (67.5)	27 (33.3)	54 (66.7)	
Sun exposure				
> 15 min/day	65 (54.2)	17 (26.2)	48 (73.8)	0.04*
≤ 15 min/day	55 (45.8)	24 (43.6)	31 (56.4)	
Everyday use of sunscreen				
Yes	46 (38.3)	19 (41.3)	27 (58.7)	0.19
No	74 (61.7)	22 (29.7)	52 (70.3)	
Current smoker				
Yes	14 (11.7)	2 (14.3)	12 (85.7)	0.14
No	106 (88.3)	39 (36.8)	67 (63.2)	
Regular exercise				
Yes	36 (30)	10 (27.8)	26 (72.2)	0.33
No	84 (70)	31 (36.9)	53 (63.1)	

Data are presented as number (percentage), * = *P*-value < 0.05, ** = *P*-value < 0.01

**Table 3:** Associated risk factors of vitamin D deficiency using binary logistic regression

Variable	Odds ratio	95% CI	*P*-value	Goodness of Fit test
Age < 45 years	3.5	1.54 to 7.98	0.003**	P = 0.3
Sun exposure ≤ 15 min/day	2.38	1.05 to 5.26	0.038*	
Current smoker	0.23	0.04 to 1.24	0.09	
Constant	0.14	0.03 to 0.53	0.004	

* = *P*-value < 0.05, ** = *P*-value < 0.01

## Discussion

The current study is the first to have assessed vitamin D status in chronic MPS patients in Thailand. The key results showed that high prevalence of vitamin D inadequacy was found among these subjects. Additionally, this study found that younger age (< 45 years) and low sunlight exposure (≤ 15 min/day) were associated with vitamin D deficiency.

An unexpectedly low percentage of subjects (34.2%) in this study were classified as deficient in vitamin D, which is comparatively less than studies from other regions that ranged from 74 to 93% [12–14]. Perhaps this could be explained by people in Thailand typically having more sunlight exposure than those in the United States, Turkey, or Saudi Arabia. However, this study found a higher prevalence of vitamin D deficiency compared to the national survey of vitamin D status in Thailand conducted by Chailurkit et al. Their survey reported that among 280 people living in Bangkok, 50.3% had vitamin D insufficiency, and 14.3% had vitamin D deficiency [23]. The present study showed that chronic MPS patients with younger age were more likely to have vitamin D deficiency which is similar to the study by Chailurkit [23]. A possible explanation is that the elderly have more free time and prefer to spend it outdoors, while young adults in Thailand tend to work indoors. Furthermore, young adults often consume low vitamin D-containing diets such as fast food and soft drinks which would explain their low vitamin D intake [24].

The SF-36 bodily pain score and the EQ-5D-5 L health utility score were not significantly correlated with serum vitamin D concentration. This is in contrast with other studies that have found vitamin D levels to be significantly correlated with quality of life [24–27]. However, most of these studies were conducted in known vulnerable populations, such as the elderly, depressed, fibromyalgia and other patients. Another possible explanation for no correlation between vitamin D and pain or health scores is that the majority of patients in the present study had received treatment before participating in the study.

This study had several strengths. Firstly, it was conducted in Thailand, where seasonal variation in the number of daytime sunlight hours is low. Hence, the whole study period had consistent conditions for sunlight exposure, which is considered to be the major source of vitamin D. Secondly, the effect of potential confounders was mitigated by performing multivariable statistical analysis with a logistic regression model. Thus, the results of this approach can be viewed with greater confidence than simple univariable analysis.

This study also had some limitations that should be considered when interpreting its results. First, data collection for this study did not include dietary vitamin D intake. Although food is not the primary source of vitamin D, this issue could potentially affect the validity of the results. Owing to the use of a cross-sectional design, risk factors associated with vitamin D deficiency cannot imply causation. To attempt to establish causal relationships would require vitamin D measurements before and after modifying risk factors. Additionally, although the sample size was calculated appropriately, the prevalence result might not be as precise as expected. This issue was due to the substantial difference between the prevalence (93%) used in the calculation formula and the observed prevalence (34%). The low prevalence also had an impact on the statistical power of the regression model potentially causing the absence of a significant association in some factors. Lastly, the research site of this study was a tertiary hospital in the capital city, central region, Thailand. The results may not be inferred to other hospital settings or other regions of the country.

## Conclusions

The current study found that approximately one third of patients with chronic MPS had vitamin D deficiency. These findings suggest that clinicians should be concerned about vitamin D deficiency among patients with chronic MPS, particularly those of a younger age and having insufficient sunlight exposure. However, vitamin D deficiency was not found to correlate significantly with reported pain or health utility scores.

## Data Availability

The datasets used and/or analysed during the current study are available from the corresponding author on reasonable request.

## Abbreviations

MPS: Myofascial pain syndrome
SF-36: Short Form-36
EQ-5D-5L: EuroQol 5-Dimension, 5-Level
